# Rationing, Responsibility, and Vaccination during COVID-19: A Conceptual Map

**DOI:** 10.1080/15265161.2023.2201188

**Published:** 2023-04-27

**Authors:** Jin K. Park, Ben Davies

**Affiliations:** a Harvard Medical School; b University of Oxford

**Keywords:** COVID-19, public health, rationing/resource allocation, pandemics, reciprocity, responsibility for health

## Abstract

Throughout the COVID-19 pandemic, shortages of scarce healthcare resources consistently presented significant moral and practical challenges. While the importance of vaccines as a key pharmaceutical intervention to stem pandemic scarcity was widely publicized, a sizable proportion of the population chose not to vaccinate. In response, some have defended the use of vaccination status as a criterion for the allocation of scarce medical resources. In this paper, we critically interpret this burgeoning literature, and describe a framework for thinking about vaccine-sensitive resource allocation using the values of responsibility, reciprocity, and justice. Although our aim here is not to defend a single view of vaccine-sensitive resource allocation, we believe that attending critically with the diversity of arguments in favor (and against) vaccine-sensitivity reveals a number of questions that a vaccine-sensitive approach to allocation should answer in future pandemics.

## INTRODUCTION

Public health ethics has long contemplated the principles that should govern the allocation of scarce resources. Many countries, including the US and UK, engage in priority-setting both at the level of health systems and at the point of care due to budgetary constraints and tradeoffs in government spending. The most controversial cases, however, cover the distribution of non-economic, “physical” (Brock 2007) resources: organ transplants; scarce experimental treatments; and, most controversially during a pandemic, ventilators and the hospital staff required to provide ICU-level care.

During the COVID-19 pandemic, such rationing has came more directly into the public eye, initially because hospitals anticipated being overwhelmed as skyrocketing case rates were followed closely by increased hospitalizations. Concerns were almost immediately raised about the possibility that rationing principles might exacerbate preexisting healthcare inequalities (Schmidt 2020; Cleveland Manchanda, Couillard, and Sivashanker 2020; Pring 2020; Scully 2020). The introduction of vaccines fundamentally reshaped pandemic responses. The initial trials of the two major mRNA vaccines provided evidence that vaccination reduces morbidity and mortality in several dimensions (Tenforde et al. 2021; Bahl et al. 2021; Grapsa et al. 2022). Despite their wide availability in wealthy countries, many people chose not to be vaccinated.

The initial uptake of vaccines followed socioeconomic, geographical, and racial gradients (Gertz et al. 2022; Saban et al. 2021; Nguyen et al. 2022; Padamsee et al. 2022). In addition, the relative risk of infection in various communities varied drastically given fluctuating case rates in localities and municipalities. As a result, a significant literature emerged during the pandemic on the choice to remain unvaccinated or partially vaccinated, and its implications for broader treatment. This paper offers a critical overview of this burgeoning literature on vaccination-based resource allocation. Our aim is not to defend a single position, but rather to map out the moral terrain in which the arguments have proceeded (c.f. Sharkey and Gillam 2010). In addition, while we draw on the events of the Covid pandemic, our aim is to develop a broader framework for thinking about vaccine-based resource allocation that will be applicable to future pandemics.

This approach will help to clarify future debates regarding vaccination and scarce resource allocation by offering a comprehensive framework for thinking about healthcare consumption in pandemic scarcity. The central question with which we are concerned, then, is whether *vaccination status* is a legitimate criterion on which to base healthcare allocation. We identify three dimensions which have been deemed relevant: the idea of responsibility; the idea of reciprocity; and the background conditions of justice. Though these are not the only important values in a pandemic, this framing helps to parsimoniously draw together a variety of arguments that have been made in this literature.

## RESPONSIBILITY AND VACCINATION

Responsibility is perhaps the key question in considering what role, if any, patients’ vaccination status should play in healthcare allocation. The idea that patients should sometimes be held responsible for their health-affecting choices has received considerable attention, including significant criticism. The strongest case made by advocates of patient responsibility for health-worsening choices requires at least two conditions to be met. First, the choice must have been foreseeably connected to increasing a patient’s health need in ways that might impact others (Davies and Savulescu 2020). Second, the choice must have been at least avoidable, and perhaps avoidably with *low cost* and *relative ease* (Davies 2020). Some critics of “responsibilization” in general have charged that our health-affecting choices do not meet these criteria, or at least that we cannot be sufficiently confident that any particular choice does so (Wikler 2002; Friesen 2018); defenders of responsibilization argue that it is at least in principle possible to fairly detect some of these choices (Savulescu 2018). The question, then, is whether the choice to remain unvaccinated meets these criteria.

Recently, Robertson (2022) has offered a harm-based argument for vaccine-sensitive scarce resource allocation, centering the responsibility that healthcare providers have to protect patients from the harm caused by other patients’ behaviors. On his view, vaccination is substantively different than other risky behaviors in being more suitable as a target of responsibility-sensitive resource allocation. For instance, Robertson compares drinking alcohol and choosing to be unvaccinated, arguing that the latter but not the former is a “proximate cause” of scarcity and is a “foreseeable risk” of that choice. This conception of vaccine responsibility has two components: (1) proximate and foreseeable cause and (2) factual tractability.

Start with the proximate and foreseeable cause condition. On this view, vaccination is foreseeably a *proximate* cause of the exacerbation of scarce resources (since being unvaccinated increases one’s risk of needing intensive care), whereas drinking alcohol is not a proximate cause of organ scarcity (even though drinking alcohol increases one’s risk of liver damage). Robertson argues that since an “unvaccinated person at the hospital demanding healthcare for COVID-19 is quite *proximately* threatening to take healthcare that would otherwise be consumed by another patient” (Robertson 2022, 11, emphasis added), vaccine refusal is morally exceptional and a suitable subject of responsibilization (Robertson 2022). The argument is familiar in other infectious disease contexts, perhaps most prominently where some have argued for liability for parents who choose not to vaccine their children, who go on to infect others (Reiss 2014; Bromberger 2017; Baxter 2014). This standard has been difficult to apply to non-vaccination during COVID-19 since absolute reduction in transmission has often been refractory to vaccination with the rise of new variants (Eyre et al. 2022). Thus, the force of this argument will vary between different pandemics depending on the effectiveness of vaccines, as well as how safe specific vaccines are for patients.

Although the proximate and foreseeable cause condition takes issue with non-vaccination in exacerbation of scarcity, other behaviors also generate “foreseeable risk of harm to others.” One potential objection to the proximate cause argument is that it does not recognize the relevance of other interventions, even when they are explicitly taken on for the explicit purpose of harm reduction. Depending on the infectious agent in question, masks and other personal protective equipment, robust social distancing and pharmaceutical interventions, as well as other measures are all tools for harm prevention by virtue of reducing individuals’ likelihood of severe illness. One challenge for those who wish to make vaccination status an allocation criterion is to explain why these other behaviors do not also count.

This brings in the second condition, factual tractability. In response to such considerations, Robertson argues that:

“[Vaccination] seems to be the only tractable criterion. An alternative, more capacious baseline would be to argue that patients must behave *reasonably* in the pandemic all-things-considered. Perhaps we would be tempted to de-prioritize a patient who attended a crowded wedding, for example? Factually, how many people attended the wedding? Was the patient closely related to the bride or groom, or just attending for fun? How good was the ventilation? When did the patient go to the event and what was the local COVID-19 case rate at the time? Did the patient wear a mask, and if so which type, and how well fitted was it? Resolving these sorts of facts would be impractical for the healthcare rationer, even if she were competent to then evaluate them…vaccination is a discrete medical decision, often present in the medical record itself, with clear and consistent public health direction about the reasonableness of undertaking the behavior.” (2022, 13).

There are several components to Robertson’s response. The first is his view that the aim of deprioritizing unvaccinated patients is to reduce harm to other patients. If we treat unvaccinated patients, this makes it more likely that we must refuse care to, and thus harm, vaccinated patients. Thus, even if it would be *better* to consider a wider range of behaviors, this is impossible in real-world medical decision-making, and it is preferable to reduce harm somewhat than not at all. On this view, those who engage in *any kind* of avoidable behavior that risks increasing scarcity (and thus harm to others) weaken their right to treatment. We might compare this argument to the idea that it is not unfair to have ticket inspectors on state-owned public transport simply because they won’t be able to catch everyone, and even if some people who have paid for their tickets have illegitimately failed to contribute to a public resource in other, less detectable ways.

Robertson’s appeal to harm, though, requires further interrogation. In one sense, any patient who uses health resources risks harming other patients, if we take a non-moralized conception of harm as simply making it more likely that others suffer some bad outcome. Thus, the notion of harm by itself will not do the required work.

A further potential issue with Robertson’s argument is that it seems to assume that, while individuals can culpably increase their risk in a variety of ways, *each way in isolation* renders one culpable regardless of how else one behaves (and it is just that vaccination status is the only way that is pragmatically detectable). But this can also be challenged. Consider Case 1:

*Megan the Cautious* is a 50-year-old retiree who expects that there is a moderate chance of serious illness or hospitalization during an ongoing pandemic, given the latest evidence from health authorities regarding her age, local case rates, and preexisting health conditions. Although she considers getting vaccinated to reduce her risk, she is concerned about possible side-effects, and instead decides to reduce her risk of infection by living alone, never leaving her home, or meeting with anyone. She nonetheless becomes ill and requires intubation and ventilation.

*Pauline the Indifferent* is a 20-year-old college student who expects that there is a moderate chance of serious illness or hospitalization during an ongoing pandemic given the latest evidence from health authorities regarding her age, local case rates, and preexisting health conditions. She is vaccinated but flouts all other public health guidance throughout multiple waves of the pandemic. She becomes ill and requires intubation and ventilation.

Pauline and Megan would be treated differently by Robertson’s proposal. But whereas we might say that Pauline has avoidably increased her risk of needing scarce resources, it is less clear that Megan has done so. Although Megan increases her risk along one dimension, this may be outweighed by the many other mitigation strategies she adopts. Robertson’s response seems to take the view that while it is fair to deprioritize the unvaccinated, it would be fairer still to deprioritize those who take on other risky behaviors too if only they were factually tractable (e.g. Robertson 2022, 13). But Megan’s case raises the issue of an individual who overall lowers their risk of needing scarce resources (compared with a baseline of doing nothing), even though they raise it along one dimension by remaining unvaccinated. In other public health contexts such as contribution to herd immunity, some argue that exemptions to vaccine mandates should only be granted to people who make an equivalent and proportional contribution to public health (Clarke, Giubilini, and Walker 2017; Giubilini, Douglas, and Savulescu 2017).

Turn now to the factual tractability condition. We might think that well-established bioethical principles give us reasons to pay particular attention to traceable sources of harm. The maxim “first do no harm” doesn’t tell us to quibble over factually indiscernible harms—it tells us that *if* there is an opportunity to avoid harm, that we must do so. Indeed, Beauchamp and Childress’s (2019, 155) principle of nonmaleficence requires healthcare workers not to impose harms or risks of harm, with the latter being governed by a standard of due care, where due care is understood “as the circumstances demand of a reasonable and prudent person.”

Advocates of vaccine-based allocation for scarce resources have defended the distinction between vaccination and other backward-looking criteria on the basis that vaccine status clearly meets the reasonable-person standard in an objective manner without tricky fact-finding operations. Persad and Largent (2022) argue that vaccine status is “readily verifiable, broadly accessible, and directly linked to the outcome of interest,” an argument similar to Robertson’s.

One challenge for the condition of factual tractability concerns the purported difference in causally tracing harm to others between vaccination and other cases, such as lung transplants and cigarette use. After all, there is hardly any dispute of the clear and causal link between cigarette use and organ scarcity, and these activities also bear physiological signs that can be ascertained.

In response, Robertson argues that in cases where clinical ethicists are resistant to use past behavior such as cigarette use as a rationing criterion, “the underlying facts are scalar (how much) rather [than] binary (yes or no), and they are difficult to ascertain” (Robertson 2022, 7). So, is the factual tractability condition committed to a binary standard of responsibility for scarcity? This will depend to some extent on the nature of the disease. Perhaps inoculating against some diseases would only require one dose, making vaccination a genuinely binary choice. But other diseases (including COVID-19) require multiple doses and boosters, with each shot contributing to a decreased risk of hospitalization and serious illness. This is especially relevant because at certain points during the COVID-19 pandemic, while racial gaps in vaccination rates in the US have closed over time, booster rates have not seen the same trends (Ndugga et al. 2022). In addition, vaccination rates do vary by ethnic group in the UK. As of July 2022, the proportion of people who remained unvaccinated among individuals of Black Caribbean ethnicity vs. White British ethnic groups was 39% and 9%, respectively (Office for National Statistics 2022). This does not mean that vaccination status is as gradable as other behaviors which have been targets of responsibilization, such as smoking; but it does raise a pragmatic challenge for what, precisely, it means to say that a patient is vaccinated or unvaccinated. We return to this question when we consider the background conditions of justice.^1^

One option here is to defend a graded account of vaccine refusal, given that treatments can be provided proportionally, and different “degrees of vaccination” tracked reliably through health records. In a different setting, unrelated to responsibility, Truog argues that one way to resolve difficult rationing problems is to divide the benefits of a ventilator equally by allowing all who need a ventilator to access it in a “time-limited trial” of therapy (Truog 2021). Advocates of this view *could* endorse providing time on ventilator in proportion to a person’s degree of vaccination. However, this would require withdrawing ventilatory support, which is frequently opposed (Liddell, Martin, and Palmer 2020).

Finally, though they do not defend the adoption of this standard, several commentators have raised the question of whether the harm caused in the decision not to get vaccinated can be analogized with harms caused in contexts such as the criminal law (GeriPal–A Geriatrics and Palliative Care Podcast 2022; Robertson 2022; Whyte and Caplan 2022). For some crimes, people can be held responsible despite a range of factors that may mitigate against moral responsibility. Some advocates of a harm-based standard suggest that we might similarly hold people *substantively* responsible for refusal to be vaccinated despite the fact that some of them will have been in circumstances that mitigate moral responsibility. Similarly, Robertson’s harm-based approach draws an analogy to drunk driving (Robertson 2022).

One challenge for this analogy to explain why this is the correct standard to apply to vaccination. In legitimate legal systems, defendants have substantive and procedural protections which may include presumption of innocence, a right to a jury trial, right to appeal, and evidentiary standards. Thus, a defense of the objective criminal standard invites the question of whether “defendants” should be provided with these other protections (see Chan et al. Forthcoming). Aside from anything else, this would significantly increase the resource costs and practicability of responsibilizing vaccination status, posing a challenge to its claim to be overall cost-saving.

### Responsibility and Vaccination in Light of Luck Egalitarianism

Broader discussions of responsibility in healthcare typically do not engage with the literature on “luck egalitarianism” (though see Robertson 2019, chap. 4), a view which (roughly speaking) endorses the idea that individual entitlements of justice can legitimately be sensitive to exercises of responsibility. Some have argued that luck egalitarianism will require vaccine-sensitive scarce resource distribution (Iserson 2022), while others have rejected entirely the idea that luck egalitarianism might offer workable insights to this problem (Robertson 2022). We believe that luck egalitarianism—and the debates that have ensued regarding the proper role of responsibility in egalitarian demands—contains important resources for thinking about responsibility for being unvaccinated, but not that it necessarily speaks in favor of vaccination-sensitivity.

Luck egalitarianism is an account of distributive equality that requires avoiding, correcting or compensating for inequalities for which agents cannot be held responsible. It standardly cuts luck into two categories: option luck and brute luck. One interpretation of this difference is that the former is the upshot of “declinable” risks and the latter is the upshot of “non-declinable” risks (Knight 2021), where a declinable risk is one the agent should have foreseen, and had the option not to take (Dworkin 1981, 293) An alternative interpretation concerns whether an outcome is “reasonably avoidable,” i.e., whether there is “for the agent, some reasonable choice that avoids that outcome” (Vallentyne 2002, 533). We do not take a position on what the best interpretation of the luck egalitarian principle is, but hope that the distinction is roughly clear for readers. Luck egalitarianism requires that individuals be compensated for inequalities that arise as a result of brute luck. Inequalities that arise due to option luck need not be compensated on grounds of equality, though this doesn’t preclude them from being compensated on the basis other values, such as beneficence, charity, or solidarity.

Consider Case 2:

*Robert the Immunocompromised* is a 40-year-old store clerk taking immunosuppressants for Inflammatory Bowel Disease who expects that there is a moderate chance of serious illness or hospitalization during an ongoing pandemic but, because of his immunocompromised status, is unvaccinated. He becomes ill and requires intubation and ventilation.

*Joe the Healthy* is a 25-year-old athlete who, because of his youth and excellent health, expects that there is a very small chance of serious illness or hospitalization during an ongoing pandemic even if he were infected, and is therefore unvaccinated. He becomes ill and requires intubation and ventilation.

*Kathy the Vaccine Refuser* is a 75-year-old retiree who, because of misinformation on the internet, expects that there is a very small chance of serious illness or hospitalization during an ongoing pandemic even if she were infected, and is therefore unvaccinated. She becomes ill and requires intubation and ventilation.

If we understand genuine choice to depend on something like “reasonable avoidability” or “declinable risk,” then perhaps we should treat Joe and Kathy’s predicament as an upshot of option luck, but Robert’s as an upshot of brute luck. For instance, Shaw (2022, 885) argues that declining a vaccination represents declining the “first and best care available” for the disease in the context of severe resource constraints, and that individuals who decline vaccination thus “[weaken] their claim to ongoing care if they become ill.” We assume that Shaw would not extend this claim to Robert, who might reasonably believe that vaccines will not work on him given widespread reports to this effect (though see Mazer 2022). But Kathy and Joe are presumably to count as refusing care by remaining unvaccinated.

Considering vaccine refusal to be a form of refusal of care is complex for two reasons. First, refusal of care in one clinical scenario is typically not treated as relevant to consideration of care in other scenarios. Some examples come close. For instance, Brown (2019) outlines how many health commissioners exclude individuals with BMIs over 30 from IVF treatment; where an individual has refused care aimed at weight loss, this *might* be seen as an example of what Shaw advocates. But even here, it is not clear, since health commissioners might still refuse IVF to an individual who has accepted weight loss treatment which has been ineffective. More broadly, considerations of responsibility typically focus on *pretreatment* behavior rather than treatment choice (though see Savulescu 2018; Davies 2020).

There are also further complications to the straightforward idea that Robert’s need results from brute luck, whereas Joe’s and Kathy’s results from option luck. The most obvious complication, from a luck egalitarian point of view, is that some will not consider Kathy’s situation to be characterized by sufficient “choice” given the influence of misinformation on her decision. According to Vallentyne (2002, 537):

“Unavoidability is at the core of the characterization of brute luck, but it seems plausible also to include (1) events for which the agent has no ability to influence the probability and (2) events for which the agent is unaware of his or her ability to influence the probability (because of false or incomplete beliefs)”

Accounts of vaccine-sensitive resource allocation recognize the moral difficulty in attributing choice to—and therefore internalizing—the decisions of fellow citizens when they act on bad or incomplete information (Robertson 2022; Claudy, Vijayakumar, and Campbell 2022), or in making choices in what Levy (2018) calls “epistemically polluted environments.” Vaccine-sensitive accounts deal with this to varying degrees, from saying that it would be “more unjust to grant such people equal priority with unvaccinated people than it would be to deprioritize them on the grounds that they refused a vaccine while in possession of imperfect information” (Shaw 2022, 889) to saying that on some level, it is irrelevant because “we do not tolerate one person harming another merely because they had good intentions or were suffering from confusion” (Robertson 2022, 17).

However, the situation of Joe and Kathy bring out further insights to vaccine-sensitive resource allocation apart from the difficulty of attributing choice. Stemplowska (2012) argues that luck egalitarians cannot only advocate choice-sensitivity, but also need to specify an “opportunity principle” concerning the range of opportunities that ought to be open to people. This can be relevant in two ways. First, many luck egalitarians stipulate that individuals should have equivalent opportunity sets in order for it to be legitimate to hold people responsible for their choices (Segall 2007; Arneson 2018). This is important in the case of vaccination due to its implications for the quality of peoples’ choices and the consequences that attach to them.

On the other hand, Stemplowska’s proposed opportunity principle is focused on when it is reasonable for others to take on the costs of individual choices. She offers an interest-based account, which asks whether the costs to an agent involved in avoiding a choice are greater than the costs involved to others in compensating for the choice-generated inequality (Olsaretti 2009, 171; Stemplowska 2009).

The cost to Joe of choosing differently than he did are arguably small; it would have been easy for him to get vaccinated, and it likely would have cost him very little. The costs of compensating the resulting inequality depend on where we focus: the costs to society as a whole of treating one patient are not that significant; but the cost may be significant for an individual who otherwise could have receive treatment (though see Witberg et al. 2021). Thus, even consideration of opportunities may still imply that we should treat the disadvantage resulting from Joe’s *decision* as stemming from a legitimate choice, in which case Joe would not have egalitarian grounds for compensation.

However, while getting vaccinated would not have cost Joe much, the chance of him becoming sufficiently unwell to need intensive care was also minimal given his circumstances.^2^ It may be unreasonable to require individuals to consider such minute risks, even if the consequences if those risks are realized would be very bad indeed. Such a perspective would be excessively demanding; stepping out the front door in the morning, we would have to be careful not to step on a crack on the sidewalk lest we flirt with the possibility of spraining our ankle and ending up in the hospital. We would need to reconsider our decision to add another pinch of salt at the dinner table, due to the small possibility that this can lead to chronic high blood pressure (Stemplowska 2016, 151).

One possible rejoinder to this is that Joe’s choice does not only represent a risk to himself; insofar as vaccines reduce not only the chance of becoming seriously ill, but also the extent to which one is likely to spread illness to others, Joe’s choice may seem to harm others in another way. Indeed, we might expand the interest account to consider not only the costs of Joe’s choosing as he does to himself, but also the costs to others. This question becomes even more complex if we think that our evaluation should focus not only on Joe’s actions in isolation, but also on what would happen if others also chose the same way (see, e.g., Stemplowska 2016, 157) Indeed, this question may be highly relevant in future pandemics where characteristics of disease transmission differ (e.g., a scenario in which vaccines offer near-sterilizing immunity), and the focus of moral responsibilization lies more squarely on transmission apart from the risk of becoming seriously ill.

However, this may conflate the egalitarian question—which inequalities should be corrected—with a *retributive* question of whether a person’s broader moral behavior can ever be relevant to their health entitlements. If the harm Joe does to others, which is only tangentially related to his current health need (both were made more likely by a common decision) is relevant, why not other morally relevant acts? (see Shaw, In Preparation). Luck egalitarians who wish to justify taking vaccination status into account may feel on more secure ground excluding the question of whether refusing to get vaccinated was *morally* suspect, and focusing on costs that would arise from the decision to *correct* the resulting inequality, rather than on broader costs that have already occurred. The fact that Joe puts others at risk in ways that cannot be avoided by a later allocation decision thus seems of little relevance to luck egalitarian conceptions of responsibility. We return to the broader harm Joe may cause when we consider the separate issue of reciprocity. Turn now to a further recent observation about the structure of luck egalitarianism.

Olsaretti (2009) notes that luck egalitarians should also articulate a “principle of stakes” that specifies what consequences should attach to various choices. This is important because consequences are sometimes socially controllable. Citizens who face unexpected loss of life or property due to unexpected natural disasters are assisted by the state despite the fact that, as some luck egalitarians have argued, living in areas known to be flood-prone could be seen as a “choice,” the upshot of which should be considered option luck (Rakowski 1991).

Some advocates of responsibility acknowledge an unfairness in the fact that there is luck involved in whether risky choices turn out badly. Deciding to remain unvaccinated increases one’s risk, but it does not necessarily mean one will become critically ill. As a response, some (Bærøe and Cappelen 2015; Cappelen and Norheim 2005) endorse holding individuals responsible for their choices, rather than the consequences of their choices. In many cases, they suggest that this may involve taxation of risky behaviors such as smoking, with income going to fund increased care. While some have similarly proposed taxing the unvaccinated (MacEachen Institute for Public Policy and Governance 2022; Chappell 2022), it is not clear whether this would be entirely analogous to taxation of smokers. The income gathered might not be able to fund increased care capacity where the main obstacle is short-term limits on physical rather than directly financial resources. Taxes on the unvaccinated seem more likely to be justified (if they can be at all) as an incentive which is less coercive than a formal mandate.

On the other hand, considering stakes might also make us wonder whether the burden associated with responsibilizing vaccination status, which may involve unvaccinated patients dying when their lives could have been saved, is proportionate or fair given the culpability of their choice. This is of course a version of the “harshness” objection to luck egalitarianism (e.g. Anderson 1999). A response to this from some luck egalitarians (e.g. Albertsen and Nielsen 2020) is that where we lack capacity to help everyone (e.g., by admitting them to ICU), it is equally, and perhaps more, harsh to allow a patient who is not responsible for their condition to suffer an increased chance of dying.

These three elements—a robust conception of choice, a rigorous engagement with opportunity sets, and a comprehensive account of the consequences that should attach to choices—are core to many sophisticated accounts of luck egalitarianism and are jointly unrecognized as tools for the question of disproportionate utilization of healthcare resources.

## VACCINATION AND RECIPROCITY

Reciprocity is critical in organized human activity, and its importance is thrown into stark relief in a global pandemic in which coordination of supply chains and essential services is strained, and the reduction of disease transmission relies on a network of overlapping commitments. Some arguments that promote a role for vaccination status in the allocation of scarce resources ground their views on the idea of reciprocity. These arguments often share a common structure, and generally proceed in one of two ways. The first takes reciprocity as an *instrumental* value that is relevant for other values in the context of scarcity. The second takes reciprocity as a *non-instrumental* value in the face of scarcity. Therefore, reciprocity has been invoked to defend vaccine-sensitive scarce resource allocation, but invocations vary in how they structure that value in relation to other values such as utility and reducing health disparities.

A recent non-instrumental account of reciprocity that might cover scarce resource allocation has been developed by Fenton (2021). Drawing on an earlier account from Becker, Fenton provides an extended treatment of the nature of pandemic reciprocity, and the scope of that value when it comes to maintaining scarce healthcare resources. Becker’s (1986) account proposes two conditions for an exchange to be considered appropriately reciprocal. The fitness condition requires that the good or service being reciprocated must be perceived by the recipient as a good. The proportionality condition requires that the return be at least as valuable as what the recipient has given in return by some metric to the recipient. This conception of reciprocity is complicated for certain goods such as a well-functioning public health system, which provides benefits that are non-excludable and in which no single individual can return the proportional benefits of the good.

It is worth noting that proportionality need not work the other way. While proportionality requires that people get out at least as much as they put in, it does *not* require that people put in at least as much as they get out: reciprocal systems are not simply “get what you give” schemes (Segall 2005). Of course, this combination is possible only when cooperation produces more value than the aggregate of individuals by themselves could have produced. In the context of the pandemic, however, we may consider a well-functioning public health system to be what Klosko (1987) calls a “presumptively beneficial” public good, which gives us demands to provide reciprocal obligations.

An anonymous reviewer notes that different healthcare systems vary in terms of how they are funded, and many are not “public” health systems. Thus, we might think that an argument for reciprocity that depends on the public nature of a health system may not apply to these cases, such as the US. However, even in systems with significant private insurance, public health responses during a major health disaster *do* tend to be publicly funded, and emergency and other preventive resources provided independently of insurance. Thus, reciprocity considerations can apply in healthcare systems with differing levels of public provision.

There are three domains that are relevant for reciprocity considerations in the maintenance of public health systems during scarcity. First, there is the question of what the scarce goods in question are. Our reciprocal obligations to maintain a functional health system are not the same as our reciprocal obligations to maintain a campsite that we have agreed to enter. Early in a pandemic, many resources may become scarce (e.g., toilet paper), but not all of them are necessary for the maintenance of the broader health system. Call this the *currency condition*. Next, there is what Fenton calls the *scope condition*: *who* is bound by the benefits and burdens of reciprocity?^3^ For instance, many have argued that in the context of public health, agents’ obligations of reciprocity do not fit neatly along state borders (Jecker 2022; Liu, Salwi, and Drolet 2020). Finally, there is the *proximity condition* of reciprocity: even if we settle the questions of object and scope, we still have the further question of which behaviors should count as proper reciprocation, and how closely connected they need to be to the reciprocal result.

Reciprocity raises the question of motivation. Becker’s test for reciprocity—that an agent fulfills their reciprocal obligations if their behavior is “appropriate as to type and quality” (Becker 1986, 106; Fenton 2021, 5)—seems agnostic regarding the motivational state of agents when engaging in acts of reciprocity. For most reciprocity-promoting behaviors, the standard for meeting reciprocity is often an “objective” standard (i.e., we assess peoples’ objective contributions) as opposed to a “subjective” attitudinal standard.

However, the question of subjective motivation has come up in previous debates regarding herd immunity for communicable diseases. Some bioethicists argue that vaccine mandates may be morally justified on grounds that they both contribute to herd immunity and reduce harm to others (Giubilini 2019, 2020; Giubilini, Douglas, and Savulescu 2018; King, Ferraz, and Jones 2022; Pierik 2018). During COVID-19, there has been debate regarding the phenomenon of herd immunity and to what extent it should be considered a reciprocal obligation. The unique biomechanics of an infectious agent can complicate the extent to which the maintenance of herd immunity can be seen as a general reciprocal obligation, since the pattern of disease transmission, the availability and effectiveness of a vaccine, and other background factors can all impact herd immunity. An appeal to reciprocity could be seen as justifying vaccine-sensitive resource allocation on the basis of two claims: (1) that vaccine refusers fail their reciprocal obligations and (2) that failure of reciprocal obligations in this context justifies a lower treatment priority for scarce resources. We take both claims in turn.

One way to characterize the claim that those who refuse vaccination fail reciprocal obligations is to consider whether vaccine refusal is a free-rider problem. Bradley and Navin (2021) argue that one reason why vaccine refusal is seen as a free rider problem is because the subjective motivations of most refusers are misunderstood; simply stated, vaccine refusers do not believe that vaccines are effective in fostering individual immunity or contribute to collective immunity, whereas, say Bradley and Navin, to be a free rider one has to see oneself as benefitting from a collective public good.

This argument could be resisted on several grounds. First, canonical definitions of free riding do not require any particular motivation (Hardin and Cullity 2020). Second, even if we accept the definition of free riding, all this means is that we face another category of individuals (free riders*) who meet the definition of free riding apart from the motivational component; there is still an open question whether free riding* warrants deprioritization. Finally, if we are interested in motivation, we might be interested in other mental states. For instance, of an individual who remains unvaccinated and does not see themselves as free riding, we might ask whether they are justified in this belief. The fact that a person casts their actions as justified does not automatically make this the case.

A central question, then, is whether arguments which appeal to reciprocity have to be motivated by the idea that excluding or deprioritizing those who do not live up to reciprocal obligations requires those individuals to have *bad motivations.* Alternatives are that it is enough that individuals are *careless* in forming their motivations; or even that motivation is irrelevant because those involved in reciprocal arrangements are justified in excluding those who do not contribute (but easily could do so) irrespective of their motivations.

The second claim (that the shirking of reciprocal obligations during pandemic scarcity justifies lower treatment priority) requires additional argument. Even if an individual agreed to enter a reciprocal exchange, and a given choice constitutes a failure to discharge their obligations within that exchange, there are various things that may be done in face of that omission. Here, again, we face Olsaretti’s point that the establishment of responsibility—in this case for a failure of reciprocity—does not require any particular view of the associated stakes. Indeed, such a view forms the basis of Parker’s (2022) claim that deprioritizing the unvaccinated based on the value of reciprocity fails a test of proportionality that should structure violations of reciprocity.

Recall Case 1, which illustrates the relevance of the proximity condition. Both Megan and Pauline require the same resources, ventilators and ICU-level care. They are both engaging in some behavior that could be classified as meeting reciprocal obligations—Megan in the form of maximal risk modulation *except* vaccination, and Pauline by being vaccinated.

Do their motivations matter? Some US survey data illustrates that the majority of unvaccinated individuals reported routine mask-wearing, and that some unvaccinated Americans who also choose to wear masks consistently chose to do so specifically on the basis of reciprocity to others, and were concerned about the spread of COVID-19 (Shere et al. 2021; Sparks et al. 2022). On the other hand, it is easy to read Megan’s behavior as being entirely self-concerned: her avoidance of the vaccination due to concerns about personal safety is coupled with attempts to minimize her chances of becoming unwell. Would it make a difference if we discovered that Pauline is entirely unconcerned about others, and got vaccinated not in order to contribute to herd immunity but precisely because she wanted to live her life “normally” without becoming critically ill? And what if Megan, while motivated not to be vaccinated by concerns about her own health, felt extremely guilty about not doing so and thus isolated herself largely out of concern not to spread the virus, and less out of concern for her own safety?

So far, we have considered arguments that defend reciprocity on non-instrumental grounds. There were numerous examples throughout the COVID-19 pandemic where reciprocity-based arguments were applied to vaccination, but in an *instrumental* manner. In other words, reciprocity served as a justification for vaccine-sensitivity, but only because it serves other values.

For instance, Persad and Largent (2022) argue that reciprocity promotes two important values: benefitting people while preventing harm; and mitigating health inequities. Persad and Largent argue that the value of reciprocity counsels us to use vaccination status as a relevant consideration insofar as it enables “granting priority to individuals who have acted to protect the community or ameliorate scarcity,” considering vaccination as the operative reciprocity-contributing behavior. Recall Joe in Case 2. We suggested that luck egalitarian views of responsibility should not obviously take the broader moral status of Joe’s decision—i.e., the fact that he risked harming others through *transmission* rather than through significantly increasing his own risk of needing ICU care—into account. However, one might think that this fact could be considered under the guise of a failure of reciprocity.

However, as Case 1 suggests, as well as descriptive survey data on the characteristics of vaccine refusers (Shere et al. 2021; Sparks et al. 2022), citizens during the pandemic did not see the vaccine as the only reciprocity-centered behavior for the preservation of scarce health resources. One question about Persad and Largent’s argument—which maps onto questions about motivation—is whether their stipulation that individuals “acted to protect the community or ameliorate scarcity” should be given a *de dicto* or *de re* reading. On a *de dicto* reading, individuals had to explicitly see themselves as aiming at community protection and/or relief of scarcity. On a *de re* reading, though, it is enough that individuals act in ways that *in fact* contribute to these goals, even if they were not their motivations. And again, Case 1 might make us wonder why we should focus on one particular kind of protective behavior: on a vaccination-focused view, Pauline’s choice (to get vaccinated) counts where Megan’s various choices do not, even if Megan’s decisions have a greater, cumulative protective effect.

## RESPONSIBILITY IN LIGHT OF UNJUST BACKGROUND CONDITIONS

As we have seen, advocates of using vaccination status as an allocation criterion typically appeal to responsibility and/or reciprocity, and the thought is that the decision to remain unvaccinated is a good candidate for a choice which individuals could, fairly easily, have made differently. Yet pandemics do not arise in a vacuum, and nor does an individual’s decision to get, or not get, vaccinated. Preexisting social and economic inequalities and the unequal burden of disease they create contribute to the shape of pandemics, and are in turn shaped by our response to them. When pandemics begin, both health-related and non-health-related resources and capabilities are unequally distributed to begin with.

It is now widely-recognized that the basic structure of society greatly impacts health outcomes (Marmot 2004). Unequal distribution of resources and capabilities generates unjust healthcare disparities when there is an “unjust distribution of the socially controllable factors affecting population health” (Daniels, Kennedy, and Kawachi 1999). Previous work has sought to clarify the conditions under which reducing health inequalities may be in tension with improving overall population health, and the conditions under which reducing health inequities should *prima facie* take precedence (Daniels 2019). This is what Daniels calls the “unsolved rationing problem”: namely, how to balance giving priority to those unjustly worse off against maximizing aggregate health benefits. Indeed, this problem will be extremely difficult to “solve,” since it involves a balancing of equity and efficiency over which reasonable people disagree.

In general, unjust background conditions have two direct effects during a pandemic. Background injustice—income inequality, lack of workplace autonomy, etc.—create *disproportionate exposure* to a virus (or some other contagion). Background injustice also impacts individuals’ prospects of survival once they become ill, and thus generates *disproportionate health burdens*. The principle of maximizing benefits, initially heralded by some commentators as the most important value in a pandemic (Emanuel et al. 2020), was criticized by others as failing to take seriously disproportionate health burdens (Cleveland Manchanda, Couillard, and Sivashanker 2020; Davydiuk and Gupta 2021; Ballantyne et al. 2020; Johnson 2020; White and Lo 2021; Ballantyne 2020; Bagenstos 2020). This tension between these two competing values has been widely debated, including the role of race and structural racism in affecting both individuals’ exposure and health burdens (Schmidt, Roberts, and Eneanya 2022).

Background injustice is directly relevant to the use of vaccination status as an allocation criterion, because the underlying values used to defend vaccine-sensitivity (responsibility, reciprocity, etc.) will diverge in their judgements of which background conditions matter, and may also be in tension with other substantive goals such as reducing health disparities or maximizing benefits. We take each in turn.

Background injustice can affect *access* to and *trust* in vaccinations. Consider first the issue of access. The most significant disparities in access have been global, with citizens of wealthy countries having significantly greater access to vaccines (United Nations Development Program 2022). But there have also been disparities in vaccine access between different socio-demographic groups in wealthy countries. Various factors which track socio-economic status may influence how easy it is to get vaccinated. Individuals with a greater amount of free time are more likely to be able to respond to last-minute invitations to get a vaccination; this will be less likely for those with considerable work or care commitments. Individuals with flexible^4^ working hours are more likely to be able to do the same, and more likely to feel comfortable risking side effects that may make them unable to work for a few days. Finally, individuals with fewer demands on their time are more likely to have the physical and mental energy to take on a non-habitual task outside of working and caring responsibilities. Proponents of vaccine-sensitive resource allocation have often analyzed the decision not to be vaccinated as a discrete *individual* moral calculus, even though as scholars have argued, anti-vaccine attitudes historically often ebb and flow with support in public institutions, funding for social welfare functions, and social solidarity (Sreedhar and Gopal 2021). Furthermore, the vaccine-sensitive resource allocation literature has sometimes omitted the unique burdens that the pandemic has placed on those who live in rural environments, including lack of testing, and health-related information barriers (Bailey, Jensen, and Ransom 2014; Perry, Aronson, and Pescosolido 2021; Mueller et al. 2021).

This is not to say that individuals who face barriers are strictly *unable* to get vaccinated. And proponents of vaccine-sensitive allocation might make two points in response. First, they may acknowledge that individuals have different levels of access and say that this should be considered in vaccine-sensitive allocation. At the very least, though, this complicates the question of what it means to have had a chance to be vaccinated. What counts as a reasonable opportunity for one person (receiving a text during working hours that a slot has opened up within the next hour) may not for another. We saw this also in discussions of the appropriate “stakes” of the choice to vaccinate (for responsibility) and the proportionality condition (for reciprocity). Moreover, this impacts the factual tractability requirement discussed earlier; while vaccination status is easy to read off a person’s medical information, the ease of access they had is not.

A second response proponents might offer is to note that there will come a point when we can reasonably say that (almost) everyone has had a reasonable chance to become vaccinated (Robertson 2022; Persad 2021). This would entail delaying vaccination-sensitivity in allocation for quite some time but may in principle avoid the problem of differential access. However, it does face a complication if a country pursues a regular booster strategy, since “full vaccination” may become a moving target from the standpoint of responsibility, which is not solved by stipulating that the responsibility threshold for vaccine-sensitive allocation comes at the first dose. Indeed, as vaccine-induced titer levels vary between individuals and can wane over time (Ward et al. 2022), it is unclear what a dosage-based threshold—as opposed to a titer-based or some other threshold (Khoury et al. 2021; Pugh et al. 2022)—captures from the point of view of responsibility, or at the very least what is linked to responsibility is shifting over time, persons, and individual circumstances.

Turn now to the issue of trust. As Razai et al. (2021) note, there was concern at various points that individuals from ethnic minorities might be less likely to become vaccinated due to a legitimate mistrust of health services and the state. This distrust may be legitimate due to past mistreatment of the individuals themselves, and due to knowledge of mistreatment of others. This may make individuals more likely to accept conspiracy theories about the dangers of vaccines, or simply to feel ill-defined unease at the thought of the vaccine. Again, such facts are not nearly as factually tractable as a simple question of vaccine status. Whether a person is mistrustful for legitimate reasons or not is a complex question that is not easily resolved even with considerable information about their personal circumstances.

Finally, vaccine-sensitive resource allocation will diverge in whether they promote or upset broader allocation principles, such as maximizing benefits or rewarding instrumental value. A purely harm-based vaccine-sensitive will upset benefit maximization, since unvaccinated patients are more likely to benefit (Robertson 2022, 19). Reciprocity-based defenses of vaccine-sensitivity may have a hard time adjudicating what to do when vaccination status goes head-to-head with being a healthcare worker, a relatively common occurrence during the pandemic (Paris et al. 2021). Graded conceptions of vaccine responsibility may help to alleviate some of these concerns insofar as they attribute responsibility to a certain point (say, the first dose) after which other criteria may be dispositive. In any case, accounts of vaccine-sensitive resource allocation should provide a sense of how to balance vaccine-sensitivity with other pro-social goals during a pandemic.

## CONCLUSION

In this paper, we have synthesized the bourgeoning literature on vaccine-sensitive scarce resource allocation. As we have demonstrated, the arguments do not live under one banner, and they invoke multiple values to either defend or oppose vaccine-sensitivity. Though we have not defended a particular view, we believe that the values that we have discussed—responsibility, reciprocity, and justice—are complex, and may be multivocal in their judgment on vaccine-sensitive scarce resource allocation. This work aims to inform ongoing scholarship regarding the role of vaccination status in scarce resource distribution.

## Footnotes

1 A further issue is that even if vaccination status is binary, patients or their families can provide incorrect information for a variety of reasons (perhaps particularly if vaccine status is known to be relevant to treatment entitlement). Robertson acknowledges this, and says that his proposal would require a universal vaccine registry.

2 We are grateful to Zofia Stemplowska for bringing our attention to this reply.

3 While Fenton discusses the “scope” of justice, she subsumes both questions of what we have termed currency, and what we have termed scope, under this idea.

4 Note that by “flexible,” we mean “within the worker’s control.” So-called “flexible” hours which are in fact unpredictable shifts given out by employers, and “flexible” jobs in the gig economy which require people to be readily available to work, are not what we have in mind.
